# 11-Oxygenated Androgens Inhibit Brown Adipose Tissue Differentiation

**DOI:** 10.1210/endocr/bqaf172

**Published:** 2025-11-19

**Authors:** Yini Yuan, Jacobie Steenbergen, Afonso de Oliveira Santos Goulart, Alba Sabaté-Pérez, Jenny A Visser

**Affiliations:** Department of Internal Medicine, Erasmus MC, University Medical Center Rotterdam, Rotterdam, 3000 CA, the Netherlands; Department of Internal Medicine, Erasmus MC, University Medical Center Rotterdam, Rotterdam, 3000 CA, the Netherlands; Department of Internal Medicine, Erasmus MC, University Medical Center Rotterdam, Rotterdam, 3000 CA, the Netherlands; Department of Internal Medicine, Erasmus MC, University Medical Center Rotterdam, Rotterdam, 3000 CA, the Netherlands; Department of Internal Medicine, Erasmus MC, University Medical Center Rotterdam, Rotterdam, 3000 CA, the Netherlands

**Keywords:** 11-oxygenated androgens, PCOS, BAT, metabolism

## Abstract

**Context:**

Women with polycystic ovary syndrome (PCOS) and PCOS animal models have diminished brown adipose tissue (BAT) activity, potentially contributing to metabolic dysfunction. Besides classical androgens, adrenal 11-oxygenated androgens are elevated in women with PCOS. However, it remains unknown whether these 11-oxygenated androgens affect BAT metabolism.

**Objective:**

To study the effects of 11-ketotestosterone (KT) and 11-ketodihydrotestosterone (KDHT) on BAT metabolism.

**Methods:**

The female mouse brown adipocyte cell line T37i was treated with increasing concentrations (0.1-10 µM) of testosterone (T), dihydrotestosterone (DHT), KT, or KDHT during or after differentiation. In addition, female mice received a daily injection of vehicle, DHT, KT, or KDHT (100 µg) for 1 day or 1 week. Adipose depots were collected for RNA sequencing (RNAseq) analysis and Gene Set Enrichment Analysis (GSEA).

**Results:**

During differentiation, T, KT, DHT, and KDHT treatment of T37i cells dose-dependently reduced lipid droplet accumulation, and downregulated mRNA expression of adipogenic markers by up to 50%, with KDHT having the weakest effect. In mature T37i cells, only the high concentrations of these androgens exhibited inhibitory effects. RNAseq analysis revealed that DHT exposure induced the most differentially regulated genes in BAT, followed by KT and KDHT treatment. GSEA indicated that 1-day treatment with DHT and KT, but not KDHT, resulted in the downregulation of metabolic pathways in BAT.

**Conclusion:**

11-Oxygenated androgens at high concentrations directly inhibit brown adipocyte differentiation in vitro and KT acutely downregulates BAT metabolic transcriptome in vivo, a result not observed with KDHT. These findings suggest that elevated 11-oxygenated androgens may impair BAT function, contributing to metabolic complications associated with hyperandrogenic conditions, including PCOS.

Polycystic ovary syndrome (PCOS) is the most common endocrine disorder in women of reproductive age, with a prevalence ranging from 5% to 18% worldwide (1). The etiology of PCOS remains unknown, although studies suggest a role for genetic, epigenetic, and environmental factors, such as environmental pollutants, diet, and gut microbiota (2, 3). PCOS not only manifests with various reproductive problems, such as menstrual irregularity and infertility, but also with metabolic complications, such as obesity, insulin resistance (IR), type 2 diabetes, and cardiovascular disease (4). According to the Rotterdam criteria, PCOS is diagnosed by the presence of at least 2 of the following 3 criteria: hyperandrogenism, oligo-ovulation or anovulation and polycystic ovarian morphology (5, 6). Hyperandrogenism is considered a hallmark of PCOS (7), being not only a diagnostic marker but also a major driver of the reproductive and metabolic symptoms observed in PCOS. Studies in female rodents and nonhuman primate models of PCOS have shown that prenatal and postnatal androgen exposure causes hypothalamic-pituitary-ovarian dysfunction (8-10). Furthermore, androgen exposure also leads to metabolic dysfunction in these models, including altered adipokine secretion, IR, and subsequent compensatory hyperinsulinemia, which exacerbates ovarian androgen excess (11-13). The advantages and limitations of these animal models and their PCOS-like traits are reviewed by Stener-Victorin et al (14).

Adipose tissue dysfunction is considered to play an important role in the pathogenesis of PCOS (15-17). Two main types of adipose tissue can be distinguished, white adipose tissue (WAT) and brown adipose tissue (BAT) (18). WAT primarily stores energy while BAT is crucial for maintaining the core body temperature by generating heat, thus increasing energy expenditure (19). Unlike unilocular white adipocytes with few mitochondria, brown adipocytes in BAT contain multilocular lipid droplets and abundant mitochondria, and highly express uncoupling protein 1 (UCP1), which is essential for nonshivering thermogenesis (20, 21). In addition, beige adipocytes, which are inducible brown-like adipocytes in WAT in response to cold exposure or other thermogenic stimuli, also express UCP1 and thus possess thermogenic potential (22, 23). Activated BAT, as well as beige adipocytes, consumes lipids via thermogenesis, and has a positive effect on energy metabolism. Several studies have reported that women with PCOS have reduced BAT mass and activity (24-26). Moreover, supraclavicular skin temperature, as a proxy for BAT activity, is negatively associated with testosterone (T) levels in women with PCOS (26). Animal models of PCOS similarly indicate decreased BAT activity correlated with hyperandrogenism (27, 28). Therefore, BAT dysfunction is suggested to contribute to the reproductive and metabolic PCOS phenotypes. In support, BAT transplantation and BAT activation upon cold exposure in androgen-induced rodent models of PCOS have been shown to reverse PCOS-related phenotypes, such as menstrual irregularity and IR, partially through the restoration of adiponectin levels (28-30). In vitro studies further support the inhibitory effects of androgens on BAT metabolism. For example, T treatment of cultured brown adipocytes decreases lipid droplet accumulation and UCP1 expression after norepinephrine (NE) stimulation (31, 32). Similarly, dihydrotestosterone (DHT) dose-dependently inhibits differentiation and mitochondrial respiration of brown adipocytes (33).

The ovaries are considered the main source of androgen excess in PCOS (34, 35), and hence research has mainly focused on the role of classic androgens in the pathophysiology of PCOS. However, the adrenal glands have also been suggested to contribute to the hyperandrogenism in PCOS, evidenced by elevated levels of dehydroepiandrosterone sulfate, a product specifically synthesized by adrenals (36). Furthermore, adrenal involvement in the endocrine-metabolic dysfunction of PCOS has gained renewed interest with recent studies identifying elevated levels of 11-oxygenated androgens in women with PCOS (37, 38). The adrenals secrete significant amounts of the precursor 11β-hydroxyandrostenedione (11OHA4) (39), which is converted by specific enzymes, such as aldo-keto reductase 1C3 (AKR1C3) and 11β-hydroxysteroid dehydrogenase type 2 (HSD11B2), in peripheral tissues, including adipose tissues (40), into biologically active 11-oxygenated androgens such as 11-ketotestosterone (KT) and 11-ketodihydrotestosterone (KDHT). The precise mechanism driving excess adrenal androgens in PCOS has not been elucidated, although an increased hyperresponsive response to adrenocorticotropin has been suggested (41, 42). In addition, insulin may directly stimulate the adrenal cortex to produce 11-oxygenated androgens (43). Since under obese and insulin resistance conditions AKR1C3 expression in adipose tissue is increased (44), this may result in a feed-forward mechanism contributing to the elevated 11-oxygenated androgens in PCOS. Indeed, numerous studies have shown that levels of these 11-oxygenated androgens correlate with metabolic risk markers, including body mass index and homeostasis model assessment of IR (38, 45-47).

It remains unknown whether 11-oxygenated androgens, similar to classic androgens, contribute to the BAT dysfunction observed in PCOS. Therefore, in this study, we investigated the effects of KT and KDHT on BAT metabolism. Using a brown preadipocyte cell line, we found that these 11-oxygenated androgens inhibit brown adipocyte differentiation, particularly at high concentrations. In addition, short-term treatments with these 11-oxygenated androgens of female mice downregulate thermogenic BAT and inguinal WAT (iWAT) metabolic transcriptome. Collectively, these findings suggest that elevated 11-oxygenated androgens also contribute to reduced BAT and iWAT thermogenic activity and associated metabolic dysfunctions in PCOS.

## Materials and Methods

### In Vitro Adipocyte Culture

The brown preadipocyte cell line T37i (RRID:CVCL_S893) (a gift from Dr Lombès, Inserm U1185, France) was cultured in standard medium and differentiated in differentiation medium for 8 days, as previously described with slight modifications (48). For differentiation experiments, cells were seeded at a density of 4 × 10^4^ cells/well in 12-well plates coated with 0.1% gelatin (G9391, Sigma-Aldrich). On reaching confluency, cells were induced to differentiate in medium containing 2 nM triiodothyronine (T3, 642511, Sigma-Aldrich) and 20 nM insulin (I1882, Merck Life Science) for 8 days.

To study the effect of androgens on the differentiation of T37i cells, cells were differentiated for 8 days in steroid-deprived differentiation medium supplemented with increasing concentrations (0.1-10 µM) of KT (A6720, Steraloids Inc), KDHT (20200, Cayman Chemical), or ethanol (EtOH) as vehicle. T (15645, Cayman Chemical) and DHT (15874, Cayman Chemical) served as positive controls for KT and KDHT, respectively. To prepare the steroid-deprived medium, 10% fetal bovine serum (FBS) was replaced by 9% dextran-coated charcoal-treated FBS (DCC-FBS) and 1% FBS, following a previously described protocol for DCC-FBS preparation (49). At the end of the differentiation period, cells were collected for further analysis.

In separate experiments, T37i cells were differentiated in the presence of 10 µM T, KT, DHT, or KDHT. On day 8 of differentiation, cells were subjected to a 3-hour starvation in starvation differentiation medium, with 10% FBS replaced by 0.2% DCC-FBS, as described previously (49). Next, cells were stimulated with 1 µM NE or hydrochloric acid as vehicle in starvation medium for 6 hours.

To assess androgen effects on mature brown adipocytes, T37i cells were first differentiated until day 7, followed by 3-hour steroid-starvation in starvation differentiation medium. Next, cells were exposed to increasing doses of T, KT, DHT, KDHT (0.1-10 µM), or EtOH as vehicle in starvation differentiation medium for 24 hours before collection for further analysis.

### BODIPY Staining

To assess intracellular lipid accumulation, differentiated T37i cells were washed with phosphate-buffered saline (PBS) and stained with 2 μM BODIPY 493/503 (D3922, ThermoFisher) in FBS-free medium for 15 minutes at 37 °C. Next, cells were washed once with PBS and maintained in standard medium. The BODIPY fluorescence intensity was measured at 490/536 nm using the CLARIOstar Plus microplate reader (BMG Labtech) using the well matrix scan mode (30 × 30).

### RNA Isolation and Quantitative PCR

Total RNA from T37i cells was extracted using the RNeasy Plus Mini kit (74136, QIAGEN) following the manufacturer’s recommendations. RNA purity and quantity were measured with a NanoDrop 8000 spectrophotometer (Thermo Scientific). cDNA was synthesized from 1 µg RNA using the Transcriptor high-fidelity cDNA synthesis kit (5081963001, Roche Diagnostics).

Quantitative PCR (qPCR) reactions were performed using 5 ng cDNA and 1 pmol primers per sample using the FastStart Universal SYBR Green Master (Rox) (4913914001, Roche Diagnostics) on a QuantStudio 7 flex real-time PCR system (Applied Biosystems, Life Technologies). Relative expression levels were calculated using the ΔΔCt method with housekeeping genes as internal controls for normalization. The primer sequences are listed in Table 1.

**Table 1.** Primer sequences

Gene	Full name	Forward primer	Reverse primer
*Actb*	Actin, β	AAGGCCAACCGTGAAAAGAT	GTGGTACGACCAGAGGCATAC
*Adipoq*	Adiponectin	GCACTGGCAAGTTCTACTGCAA	CTAGGTGAAGAGAACGGCCTTGT
*Apoa2*	Apolipoprotein A-II	TGGTCGCACTGCTGGTAAC	TTTGCCATATTCAGTCATGCTCT
*Ar*	Androgen receptor	TCCAAGACCTATCGAGGAGCG	GTGGGCTTGAGGAGAACCAT
*B2m*	β2 Microglobulin	ATCCAAATGCTGAAGAACGG	CAGTCTCAGTGGGGGTGAAT
*Cacna1c*	Calcium channel, voltage-dependent, L type, α 1C subunit	ATGAAAACACGAGGATGTACGTT	ACTGACGGTAGAGATGGTTGC
*Cand2*	Cullin associated and neddylation dissociated 2	GTCCAGCGACAAAGACTTCAG	TCCACAATGTTCTCCACTTGGTA
*Cebpb*	CCAAT/enhancer binding protein β	ACGACTTCCTCTCCGACCTCT	CGAGGCTCACGTAACCGTAGT
*Cidea*	Cell death–inducing DNA fragmentation factor, α subunit-like effector A	AAACCATGACCGAAGTAGCC	AGGCCAGTTGTGATGACTAAGAC
*Hprt*	Hypoxanthine phosphoribosyltransferase 1	GCAGTACAGCCCCAAAATGG	AACAAAGTCTGGCCTGTATCCAA
*Il33*	Interleukin 33	TCCAACTCCAAGATTTCCCCG	CATGCAGTAGACATGGCAGAA
*Lsm3*	LSM3 homologue, U6 small nuclear RNA and mRNA degradation associated	ATGGCGGACGACGTAGATCA	AGCTCTCGGTCATTTCTCATTTT
*Nr4a1*	Nuclear receptor subfamily 4, group A, member 1	TTGAGTTCGGCAAGCCTACC	GTGTACCCGTCCATGAAGGTG
*Pparg2*	Peroxisome proliferator–activated receptor γ 2	CTCTGTTTTATGCTGTTATGGGTGA	GGTCAACAGGAGAATCTCCCAG
*Ppargc1a*	Peroxisome proliferative–activated receptor, γ, coactivator 1 α	CCCTGCCATTGTTAAGACC	TGCTGCTGTTCCTGTTTTC
*Prdm16*	PR domain containing 16	GACATTCCAATCCCACCAGA	CACCTCTGTATCCGTCAGCA
*Ttc38*	Tetratricopeptide repeat domain 38	CTGGGCTCCCACTCTCAAC	GGTCCACTTGACGTACTGAGTC
*Ucp1*	Uncoupling protein 1	GGCCTCTACGACTCAGTCCA	TAAGCCGGCTGAGATCTTGT

### Protein Extraction and Western Blot

Protein extracts were prepared by lysing cells in RIPA buffer (89900, ThermoFisher) containing inhibitor cocktails of proteases (11836170001, Roche Diagnostics; 1:100) and phosphatases (P5726, Sigma-Aldrich; 1:100). Lysates were sonicated using a small probe (Soniprep 150, MSE) and centrifuged at 13 000 rpm for 15 minutes. Supernatants were collected and protein concentrations were quantified relative to the BSA standard (10735086001, Sigma-Aldrich) using the BPA reagent (5000006, Dye Reagent, BioRad).

Western blot analysis was performed as described previously (48), with 30 μg protein extracts loaded onto 10% bis-tris precast gels (MP10W10, Millipore). After electrophoresis and transfer, nitrocellulose membranes were stained using Revert 700 Total Protein Stain Kit (926-11011, LICOR) and scanned at a wavelength of 700 nm on an Odyssey imaging system (LI-COR, Biotechnology). Next, membranes were destained, blocked in 3% skim milk in PBS, and incubated overnight at 4 °C with the following primary antibodies: UCP1 (Abcam catalog No. ab10983, RRID:AB_2241462; 1:1000), PPARG (Cell Signaling Technology catalog No. 2443, RRID:AB_823598; 1:1000), total OXPHOS antibody cocktail (Abcam catalog No. ab110413, RRID:AB_2629281; 1:500), or Vinculin (Santa Cruz Biotechnology catalog No. sc-73614, RRID:AB_1131294; 1:10 000), diluted in Intercept (PBS) blocking buffer (927-70001, LI-COR) supplemented with 0.1% Tween 20 (P1379, Sigma-Aldrich). Following wash steps with 0.1% Tween 20 in PBS, membranes were incubated with either goat-anti-rabbit DyLight 800 secondary antibody (Thermo Fisher Scientific catalog No. SA5-35571, RRID:AB_2556775; 1:10 000) or goat-anti-mouse DyLight 800 secondary antibody (Thermo Fisher Scientific catalog No. SA5-35521, RRID:AB_2556774; 1:10 000) in PBS containing 3% skim milk and 0.1% Tween 20. Immunoreactivity was measured using an Odyssey imaging system and quantified with Image Studio Lite software (version 5.2, LI-COR).

### Mitochondrial Mass and Potential Measurement

Mitochondrial mass was measured using MitoTracker Green (M7514, Invitrogen), a selective probe that covalently binds to mitochondrial proteins and accumulates in the mitochondrial matrix (50). Mitochondrial potential was assessed using the fluorescence dye, tetramethylrhodamine, methyl ester (TMRM, T5428, Sigma-Aldrich). Mature T37i cells, differentiated in the presence of 10 µM T, KT, DHT, KDHT, or EtOH, were washed with PBS and stained with 100 nM MitoTracker Green or 200 nM TMRM in standard medium for 30 minutes at 37 °C in the dark. Next, cells were washed with PBS and gently detached with TrypLE (11598846, ThermoFisher) for 5 minutes. The reaction was stopped by adding an equal volume of standard medium.

For flow cytometric analysis, cells stained with MitoTracker Green were excited using a laser at 495 nm and emission measured at 519 nm through a fluorescein isothiocyanate (FITC) filter. The average fluorescence intensity was calculated from a total of 10 000 gated events to determine mitochondrial mass for each condition by flow cytometry (BD Accuri C6 Plus, BD Biosciences). The data analysis was performed using BD software (BD Biosciences).

To determine the non–mitochondrial-dependent potential, cells were treated with increasing concentrations of carbonyl cyanide-p-trifluoromethoxyphenylhydrazone (FCCP, C2920, Sigma-Aldrich, 1-50 µM) alongside TMRM staining. Mitochondrial potential was evaluated with excitation at 565 nm and emission at 565 nm through a phycoerythrin (PE) filter by flow cytometry. The non–mitochondrial-dependent potential was defined as the lowest TMRM fluorescence intensity level induced by FCCP treatment, and this value was subtracted from the mitochondrial potential measurements in FCCP-untreated cells. The potential per mitochondrion was calculated by dividing the adjusted mitochondrial potential by mitochondrial mass.

### Animals, Housing Conditions, and Androgen Treatment

The animal experiment was approved by the animal ethics committee of Erasmus MC, Rotterdam, the Netherlands (174317-04). Female 16-week-old C57BL/6 mice, obtained from wild-type breeding strains, were individually housed under standard conditions at room temperature (∼22 °C) with a 12-hour light/12-hour dark cycle. Mice had ad libitum access to chow food pellets (801722 CRM(P), Special Diets Services) and drinking water, along with nesting materials and woodchip bedding.

After a 1-week acclimatization period, groups of 4 female mice received a subcutaneous injection of 100 µL vehicle (sesame oil), 100 µg KT, or 100 µg KDHT (each dissolved in 100 µL vehicle) at the dorsal neck region. As we previously showed that DHT reduced the expression of thermogenic markers in BAT in female mice treated with DHT for 1 week and in a DHT-induced mouse model of PCOS, an equivalent dose of DHT was administered to a fourth animal group, serving as a positive control (13, 51). This concentration was selected based on previous studies in male and female mice, in which it was shown that 100 µg was the minimum dose required to restore prostate size in castrated male mice and induce an uterine transcriptional response in female mice (52-54). The injections were administered either as a single dose (1-day treatment) or daily for 1 week (1-week treatment).

Mice were weighed prior to the first and after the last injection (1-day or 1-week). Blood and tissues samples were collected 24 hours after the corresponding final injection. Mice were euthanized by cardiac puncture under isoflurane (B506, Zoetis) anesthesia. Blood samples were immediately placed on ice. Tissues were dissected, weighed, and a piece was snap-frozen in liquid nitrogen. A separate piece of the adipose depots was fixed in 4% paraformaldehyde (P6148, Sigma-Aldrich) in PBS (14190, Gibco) at room temperature for histological analysis.

### Library Preparation, RNA Sequencing, and Bioinformatics Analysis

Total RNA from BAT was isolated with Tripure isolation reagent (11667165001, Roche Diagnostics) according to the manufacturer’s instructions, and RQ1 RNase-free DNase (M6101, Promega) was used to diminish genomic DNA contamination. Total RNA from each sample was checked for quality on an Agilent Technologies 2100 Bioanalyzer (G2938B, Agilent Technologies) using an RNA nano assay. RNA sequencing (RNAseq) libraries were prepared according to the Illumina TruSeq stranded mRNA protocol (www.illumina.com). Briefly, 200 ng of total RNA was purified using poly-T oligo-attached magnetic beads to capture poly-A–containing mRNA. The poly-A–tailed mRNA was fragmented, and cDNA was synthesized using SuperScript II and random primers in the presence of actinomycin D. The cDNA fragments were end-repaired, purified with AMPure XP beads, and A-tailed using Klenow exo-enzyme in the presence of dATP. Paired-end adapters with dual index (Illumina) were ligated to the A-tailed cDNA fragments, and then purified using AMPure XP beads. The resulting adapter-modified cDNA fragments were enriched by PCR using Phusion polymerase as follows: 30 seconds at 98 °C, 15 cycles of (10 seconds at 98 °C, 30 seconds at 60 °C, 30 seconds at 72 °C), and 5 minutes at 72 °C. PCR products were purified using AMPure XP beads and eluted in 30 µL of resuspension buffer. A total of 1 µL of resulting sample was loaded on an Agilent Technologies 2100 Bioanalyzer using a DNA 1000 assay to determine the library concentration and assess quality. The sequencing libraries were pooled together to achieve a final concentration of 2 nM, with a loading concentration of 650 pM. A 1% Illumina PhiX was spiked into the library, and sequencing was performed on an Illumina NextSeq2000 platform with P3 Flowcell for paired-end 50 bp reads.

Differential expression analysis was performed in R with the function DGEList from edgeR package, on normalized gene counts, excluding genes with low count (<1 count per million in half the samples).The selection of differentially expressed genes (DEGs) was determined by a statistical significance threshold of *P* < .05. Multidimensional scaling (MDS) plots were performed with plotMDS to assess variance between groups. Additionally, a heat map was generated with pheatmap to display DEGs between control group and androgen-treated groups. Volcano plots were created to visualize comparison of gene expression levels.

### Enrichment Pathway Analysis (Gene Set Enrichment Analysis)

To analyze the statistical enrichment of DEGs, pathway analysis was conducted using Gene Set Enrichment Analysis (GSEA) with hallmark gene sets. GSEA was performed under default settings to identify the coordinated enrichment of functionally linked genes. Briefly, the GSEA algorithm traversed the preranked dataset and calculated a running enrichment score (ES) by increasing the ES value when a dataset gene matched the gene set of interest or decreasing the ES value if it did not. A gene set was identified as enriched if the final ES was high and weighted toward a specific phenotype. Nominal *P* values were then adjusted for multiple testing correction through the false discovery rate (FDR). Gene sets with an FDR < .25 and a *P* value < .05 were considered significantly regulated. The normalized ES was the primary statistic to evaluate the degree of pathway enrichment.

Functional annotation and pathway analysis of DEGs overlapping between BAT and iWAT was performed using the Database for Annotation, Visualization, and Integrated Discovery (DAVID) web tool. DAVID is a comprehensive source for the functional interpretation of high-throughput gene expression profiles (55). The list of DEGs was uploaded to the DAVID platform, and significant enriched pathways were identified based on the Kyoto Encyclopedia of Genes and Genomes database (KEGG) using a statistical significance threshold of *P* < .05.

### Adipose Tissue Histology and Quantification

Paraffin-embedded BAT and iWAT samples, fixed in paraformaldehyde, were sectioned into 5-µm-thick slices using a microtome. These sections were mounted on glass slides and stained with hematoxylin and eosin. Whole-slide scans were performed with a digital imaging system (Hamamatsu NanoZoomer 2.0HT Digital Slide Scanner). Representative images from 3 sections per animal were randomly selected and captured using viewing software (NDP.view2). Lipid area quantification and adipocyte size were performed on these images using an automated macro in Fiji ImageJ.

### Statistical Analysis

Data analyses were performed using GraphPad Prism (version 9, GraphPad Software). Unless otherwise indicated, differences between treatments were analyzed by one-way analysis of variance (ANOVA) with a Dunnett post hoc test. Data are presented as mean ± SEM, with statistical significance defined at *P* < .05. Each experiment represents at least 3 biological replicates.

## Results

### Elevated 11-Oxygenated Androgens Dose-Dependently Inhibit Brown Adipogenesis

**Figure 1.**
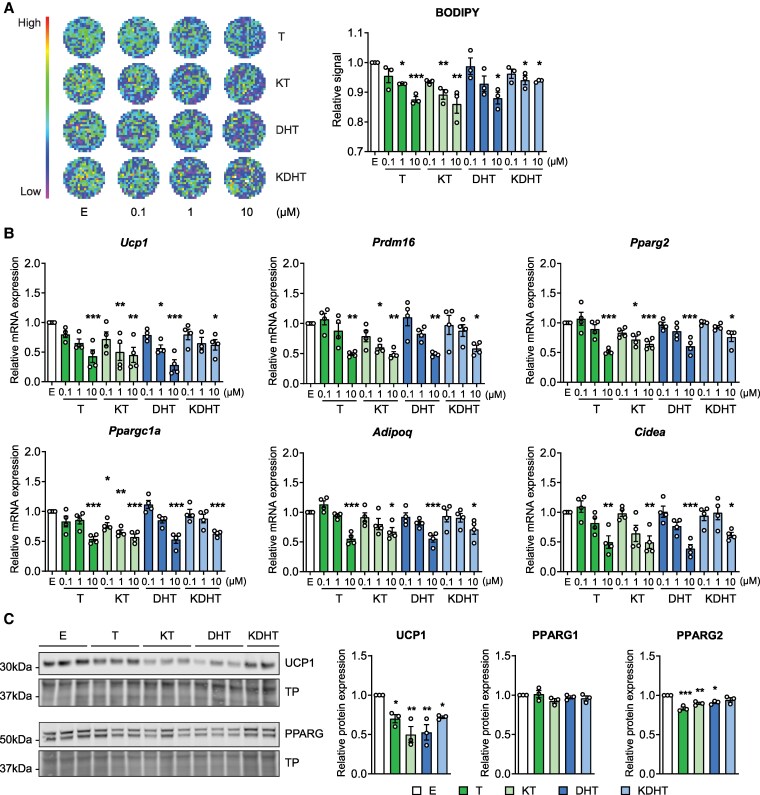
11-Oxygenated androgen treatment during differentiation suppresses brown adipogenesis. T37i cells were cultured in differentiation medium containing ethanol (EtOH; E) as control or increasing doses (0.1-10 µM) of testosterone (T), 11-ketotestosterone (KT), dihydrotestosterone (DHT), or 11-ketodihydrotestosterone (KDHT) for 8 days. A, Representative images of BODIPY staining in differentiated cells and relative signal intensity visualized and quantified with a CLARIOstar plate reader (n = 3). B, Relative mRNA expression of adipogenic markers (n = 4). C, Representative Western blot images and relative protein quantification of brown adipocyte markers UCP1 and PPARG proteins in T37i cells treated with 10 µM T, KT, DHT, or KDHT (n = 3). Data are presented as mean ± SEM. Statistical significance was determined by one-way ANOVA and indicated by *P < .05; **P < .01; or ***P < .001 compared to E control, by Dunnett post hoc test.

To determine the effect of 11-oxygenated androgens on brown adipogenesis, confluent T37i cells were differentiated in the presence of KT and KDHT at concentrations ranging from 0.1 to 10 µM. The classic androgens T and DHT served as positive controls. Consistent with previous studies (33, 56), we observed that, compared to vehicle treatment, T and DHT treatments during differentiation inhibited brown adipogenesis in a dose-dependent manner. Similarly, increasing concentrations of the tested 11-oxygenated androgens dose-dependently reduced lipid droplet accumulation in T37i cells, as assessed by BODIPY staining (Fig. 1A). Specifically, the highest concentration of KT (10 µM) resulted in an approximate 14% reduction in lipid deposition (*P* < .05), which was comparable to the effect observed with T treatment. KDHT treatment inhibited adipogenesis by only 7% (*P* < .001), which was milder than the DHT-induced reduction (Fig. 1A). In agreement, the expression of brown adipocyte markers, *Ucp1*, *Pparg2*, *Prdm16*, *Ppargc1a*, *Cidea*, and *Adipoq*, was significantly downregulated in a dose-dependent manner to maximally 50% on treatment with each androgen (Fig. 1B). Also in gene expression levels, KT induced effects comparable to T, while KDHT-induced downregulation was lower than elicited by DHT.

Western blot analysis confirmed that KT treatment at high concentration during T37i differentiation led to a 50% decrease in the protein expression of the thermogenic marker UCP1 (*P* < .01) and a 10% reduction of the adipogenic marker PPARG2 (*P* < .01) (Fig. 1C). KDHT treatment resulted in a 20% and 5% reduction of expression of these markers (*P* < .05), respectively (Fig. 1C). However, treatment with T, DHT, KT, or KDHT had no statistically significant effect on the expression of the mitochondrial proteins ATP5A, UQCRC2, SDHB, and NDUFB8 (Supplementary Fig. S1) (57). Additionally, treatment with these androgens did not decrease mitochondrial mass or the mitochondrial potential (Supplementary Fig. S1) (57).

Since the thermogenic pathway in brown adipocytes is activated in response to NE stimulation (58), we analyzed whether 11-oxygenated androgens attenuated brown adipocytes activation upon NE treatment. Following differentiation in the presence of 10 µM T, KT, DHT, KDHT, or EtOH as control, T37i cells were stimulated with NE or vehicle. As expected, NE stimulation resulted in a 12-fold upregulation of *Ucp1* mRNA levels under EtOH conditions. Interestingly, for each androgen tested, the maximal NE-induced *Ucp1* expression was comparable (Supplementary Fig. S1) (57). These findings suggest that despite the decreased differentiation state induced by the analyzed classic and 11-oxygenated androgens, brown adipocytes retain their signaling capacity in response to NE.

### 11-Oxygenated Androgens Decrease Expression of Brown Adipocyte Markers in Mature Brown Adipocytes

**Figure 2.**
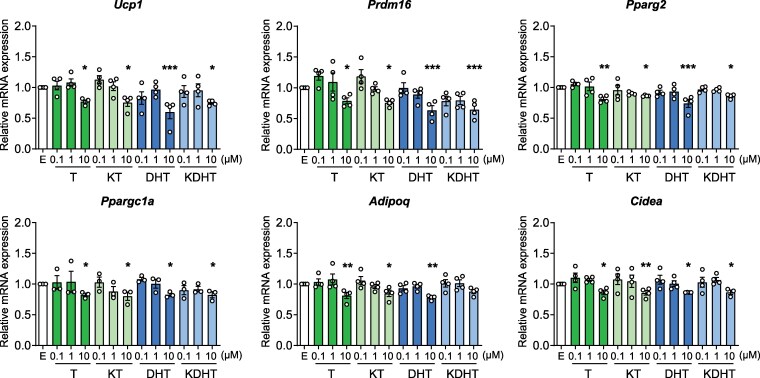
Expression of adipogenic markers is reduced by 11-oxygenated androgens treatment in mature brown adipocytes. Relative mRNA expression of adipogenic markers in differentiated T37i cells treated with 0.1-10 µM testosterone (T), 11-ketotestosterone (KT), dihydrotestosterone (DHT), 11-ketodihydrotestosterone (KDHT), or ethanol (EtOH; E) as control for 24 hours (n = 3-4). Data are presented as mean ± SEM. Statistical significance was determined by one-way ANOVA and indicated by *P < .05; **P < .01; or ***P < .001 compared to E control, by Dunnett post hoc test.

To examine the effect of 11-oxygenated androgens on mature brown adipocytes, fully differentiated T37i cells were treated with 0.1 to 10 µM T, KT, DHT, KDHT, or EtOH as control for 24 hours. In mature brown adipocytes, only the high concentration significantly reduced expression of the adipogenic markers, including *Ucp1*, *Pparg2*, *Prdm16*, *Ppargc1a*, *Adipoq*, and *Cidea*, by approximately 20% (Fig. 2). No differences were observed between the classic and 11-oxygenated androgens. These results indicate that high concentrations of the analyzed 11-oxygenated androgens also suppress the expression of brown adipocyte markers in differentiated adipocytes, albeit with a milder effect compared to brown preadipocytes.

### Identification of Differentially Expressed Genes by RNA Sequencing Analysis in Adipose Tissues of Androgen-treated Female Mice

To determine the in vivo effect of 11-oxygenated androgens, we used 2 experimental setups. First, to study the acute effects of 11-oxygenated androgens, female mice received a single injection of vehicle, DHT, KT, or KDHT (1-day treatment). Second, to examine the short-term effects of 11-oxygenated androgens, female mice received a daily injection of these androgens for 1 week (1-week treatment). Throughout the treatment period, body weight was monitored, which remained stable across all groups. Similarly, adipose tissue weight, including BAT and iWAT, did not differ between the treatment groups per treatment period (data not shown).

**Figure 3.**
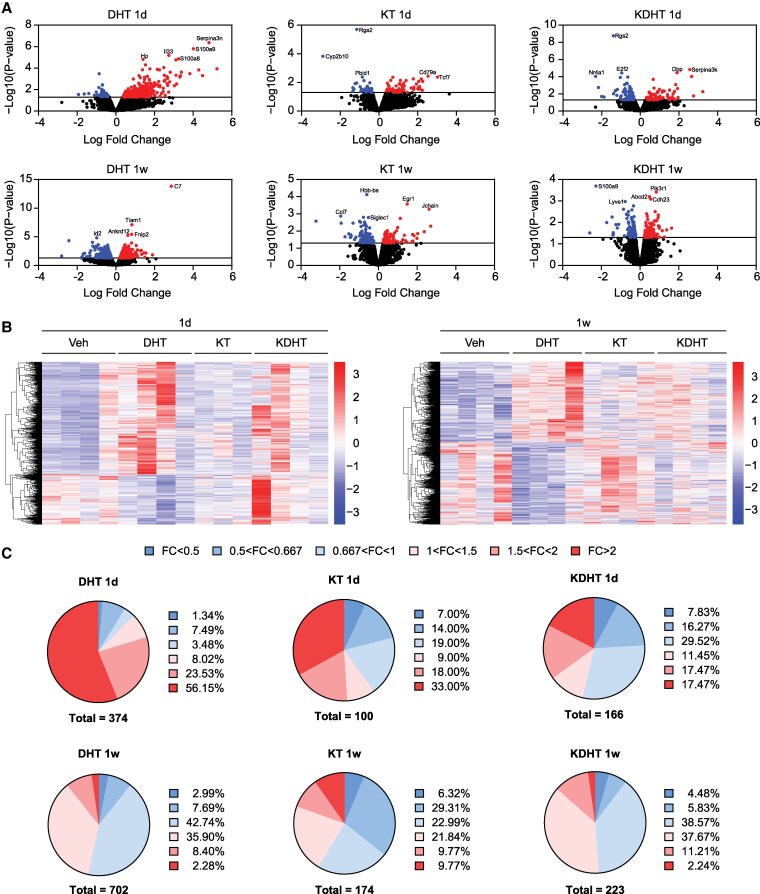
Transcriptional profiling in brown adipose tissue (BAT) of female mice treated with dihydrotestosterone (DHT), 11-ketotestosterone (KT), or 11-ketodihydrotestosterone (KDHT). Female mice received a 1-day (1d) or 1-week (1w) treatment with 100 µg/day DHT, KT, KDHT, or vehicle. A, Volcano plots display the differentially expressed genes (DEGs) on DHT, KT, or KDHT treatment for 1d or 1w. DEGs were filtered by *P* value < .05 (blue: downregulated genes; red: upregulated genes), and the top 5 DEGs based on *P* value have been labeled. B, Heat maps and dendrograms representing the DEG profiles in BAT on 1d or 1w treatment. C, Pie charts depict the number and percentage of DEGs after 1d or 1w treatment. Significant DEGs were selected with *P* value < .05 and classified based on fold change (FC) cutoffs.

RNAseq of BAT was performed to determine differentially expressed transcripts in response to the androgen treatment. iWAT was also analyzed by RNAseq as this depot contains beige adipocytes that, similar to brown adipocytes, have a thermogenic capacity. A total of 21 493 transcripts were identified, and DEGs were filtered by *P* < .05. Volcano plots with androgen-to-vehicle fold changes displayed the DEGs in BAT (Fig. 3A) and iWAT (Supplementary Fig. S2) (57), as shown in the heat map with a significant transcriptional profile in BAT (Fig. 3B) and iWAT (Supplementary Fig. S2) (57).

In the 1-day treatment group, DHT treatment resulted in 374 DEGs in BAT, of which 328 genes (87.7%) were upregulated and 46 downregulated. Of the 328 upregulated DEGs, 210 exhibited a fold change > 2 (Fig. 3C). In contrast, KT and KDHT treatment resulted in fewer DEGs in BAT (100 and 166 DEGs, respectively), with approximately equal numbers of upregulated and downregulated genes (see Fig. 3C). Similar results were observed in iWAT (see Supplementary Fig. S2) (57). In the 1-week treatment group, a greater number of DEGs in BAT were identified upon DHT, KT, or KDHT treatment, although lower numbers were again observed for KT and KDHT (174 and 223 DEGs, respectively) compared to DHT (702 DEGs). In all treatment conditions, a similar number of upregulated and downregulated genes were observed (Fig. 3C). Compared to BAT, 1-week treatment resulted in a higher proportion of androgen-induced downregulated DEGs in iWAT (Supplementary Fig. S2) (57).

### Metabolic Pathways Are Downregulated in Brown Adipose Tissue and in Inguinal White Adipose Tissue upon Dihydrotestosterone, 11-Ketotestosterone, or 11-Ketodihydrotestosterone Treatment

**Figure 4.**
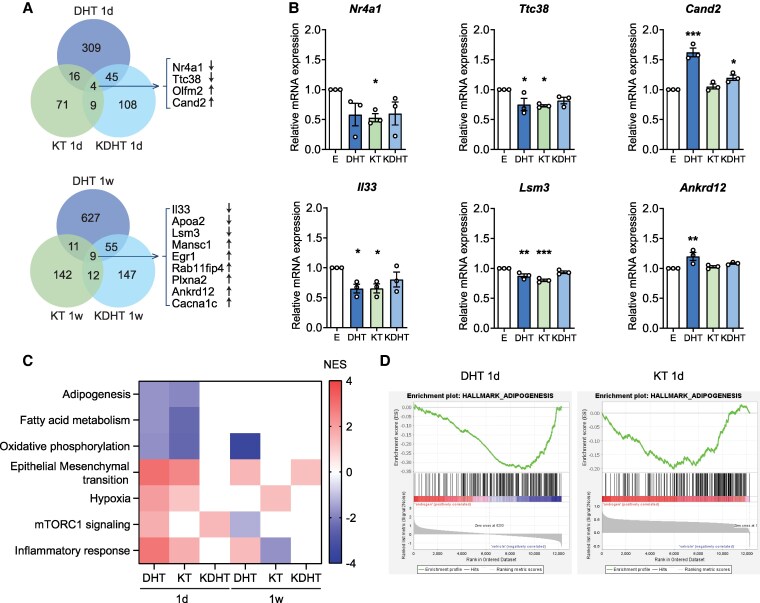
11-Oxygenated androgens downregulate metabolic pathways in brown adipose tissue (BAT). A, Venn diagrams displaying differentially expressed genes (DEGs) in BAT after 1-day (1 d) or 1-week (1 w) treatment of female mice with 100 µg/day dihydrotestosterone (DHT), 11-ketotestosterone (KT), 11-ketodihydrotestosterone (KDHT), or vehicle. B, Relative mRNA expression of identified DEGs in T37i cells treated with 10 µM DHT, KT, KDHT, or ethanol (EtOH; E) as control during differentiation. C, Heat map representing normalized enrichment score (NES) of significantly enriched pathways (FDR < 0.25 and *P* < .05) in BAT of female mice treated with 100 µg/day DHT, KT, or KDHT for 1d or 1w compared to vehicle-treated mice and determined by gene set enrichment analysis (GSEA) using hallmark gene sets. D, GSEA score curve plots of adipogenesis pathway in BAT upon 1d treatment with DHT or KT. FDR, false discovery rate. Gene expression data are presented as mean ± SEM. Statistical significance was determined by one-way ANOVA and indicated by *P < .05; **P < .01; or ***P < .001 compared to E control or vehicle, by Dunnett post hoc test.

Comparing the DEGs after 1-day treatment, only 4 genes were significantly regulated by each of the analyzed androgens. These genes were *Nr4a1*, *Ttc38*, *Olfm2*, and *Cand2* (Fig. 4A). Surprisingly, no overlapping genes were observed in iWAT (Supplementary Fig. S3) (57). Similarly, for the 1-week treatment group, 9 genes were identified in BAT that were regulated by both DHT and the analyzed 11-oxygenated androgens. These genes were *Il33*, *Apoa2*, *Lsm3*, *Mansc1*, *Egr1*, *Rab11fip4*, *Plxna2*, *Ankrd12*, and *Cacna1c* (Fig. 4A). In iWAT, we identified 6 overlapping genes, namely *Tmem98*, *Mrps6*, *Hacl1*, *Zfp882*, *Mmp12*, and *Il1b* (Supplementary Fig. S3) (57).

To validate the androgen-induced effect on the expression of these 13 overlapping DEGs in BAT, we analyzed their expression in T37i cells treated with DHT, KT, or KDHT during differentiation. Of the 13 genes, 10 were expressed in T37i cells. We found that the expression of 6 genes (*Nr4a1*, *Ttc38*, *Cand2*, *Il33*, *Lsm3*, and *Ankrd12*) was significantly upregulated or downregulated by DHT, KT, or KDHT, consistent with the in vivo results observed in BAT (Fig. 4B).

To further investigate the differential biological activity of DHT, KT, or KDHT in adipose depots, we performed GSEA. Although the overall overlap in pathways regulated by 3 treatments was limited, a subset of pathways emerged as commonly regulated, indicating diverse and shared effects between these androgens (Supplementary Tables S1-S4) (57). Following 1-day treatment, BAT exhibited significant downregulation of metabolic pathways, such as adipogenesis, fatty acid metabolism, and oxidative phosphorylation, in response to both DHT and KT (Fig. 4C and 4D). In contrast, in iWAT, only KT led to a suppression of adipogenesis and fatty acid metabolism pathways (Supplementary Fig. S3) (57). After 1-week treatment, DHT elicited a significant downregulation of the oxidative phosphorylation pathway in BAT, whereas KT or KDHT showed no significant regulation of this pathway (Fig. 4C). However, morphological analysis of BAT suggested that the 1-week treatment of DHT, KT, and KDHT tended to increase lipid droplet accumulation, indicative of reduced thermogenic activity (Supplementary Fig. S4) (57). In iWAT, the 1-week treatment of DHT, KT, and KDHT consistently downregulated these metabolic pathways, with morphological assessment indicating a tendency toward increased adipocyte size (Supplementary Fig. S4) (57).

**Figure 5.**
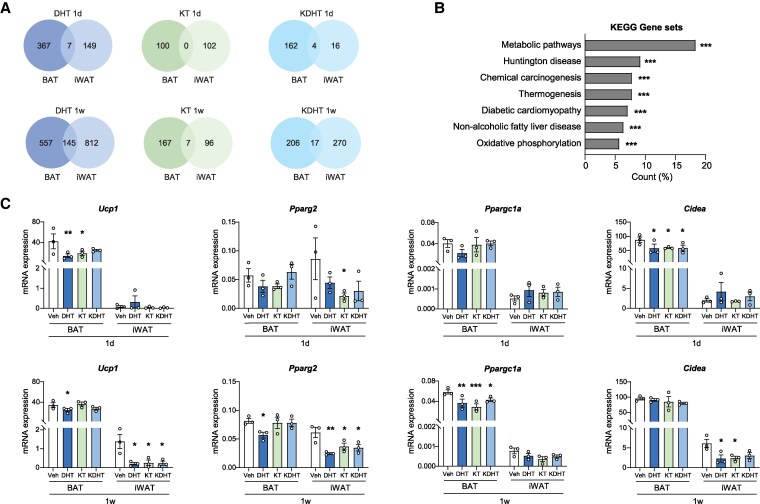
11-Oxygenated androgens decrease the expression of thermogenic genes in brown adipose tissue (BAT) and inguinal white adipose tissue (iWAT). Female mice received a 1-day (1 d) or 1-week (1 w) treatment with 100 µg/day dihydrotestosterone (DHT), 11-ketotestosterone (KT), 11-ketodihydrotestosterone (KDHT), or vehicle. A, Venn diagrams displaying differentially expressed genes (DEGs) in BAT and iWAT for each androgen treatment for 1 d or 1 w. B, Percentage of the 145 overlapping DEGs between BAT and iWAT after 1w DHT treatment represented in Kyoto Encyclopedia of Genes and Genomes database (KEGG) gene sets. C, mRNA expression of thermogenic markers in BAT and iWAT (n = 3). Statistical significance was determined by 2-way ANOVA and indicated by *P < .05; **P < .01; or ***P < .001 compared to vehicle, by Dunnett post hoc test.

In addition, a comparison analysis of DEGs between BAT and iWAT following androgen treatment revealed that only a limited number of genes were shared after DHT, KT, or KDHT treatment for 1 day or 1 week, indicating that BAT and iWAT exhibit distinct transcriptional responses to 11-oxygenated androgens (Fig. 5A). An exception was noted for the 1-week DHT treatment, showing 145 overlapping DEGs between the 2 adipose depots (Fig. 5A). Functional annotation of these 145 genes identified that they were mainly involved in metabolic pathways (18.4%) from the KEGG gene sets (Fig. 5B). Notably, a significant subset of these genes was associated with thermogenesis and oxidative phosphorylation pathways (Fig. 5B). Therefore, we measured the mRNA expression levels of thermogenic markers in both BAT and iWAT. After 1-day treatment with 11-oxygenated androgens, *Ucp1* and *Cidea* expression was significantly downregulated in BAT, whereas in iWAT, only KT induced a significant reduction in *Pparg2* expression (Fig. 5C). In contrast, 1-week androgen treatment mainly suppressed the expression of *Ucp1*, *Pparg2*, and *Cidea* in iWAT, while in BAT, only *Ppargc1a* expression was downregulated by 11-oxygenated androgens (Fig. 5C).

## Discussion

In this study, we investigate whether the newly identified 11-oxygenated androgens, which are elevated in women with PCOS, exert an inhibitory effect on BAT in vitro and in vivo. Studies have shown that women with PCOS exhibit reduced BAT activity, thus contributing to the pathophysiology of PCOS (26, 59). Indeed, activation of BAT thermogenesis in androgen-induced rodent models of PCOS alleviate the reproductive and metabolic dysfunctions (60). Previously, it has been indicated that ovarian androgens inhibit BAT activity. Here, we show that the adrenal-derived 11-oxygenated androgens also reduce BAT metabolism and thus may contribute to the pathological conditions associated with PCOS.

Brown adipocyte differentiation entails lipid droplet accumulation (lipogenesis) and mitochondrial biogenesis. During the differentiation program, ATP–consuming lipogenesis is supported by enhanced mitochondrial content (61, 62). Our findings indicate that exposure to the 11-oxygenated androgens KT and KDHT during brown adipocyte differentiation dose-dependently inhibited both lipid deposition and expression of adipogenic genes, indicating suppressed lipogenesis. In addition, these 11-oxygenated androgens downregulated the expression of thermogenic markers, including *Ucp1*, *Prdm16*, and *Ppargc1a*, suggesting a diminished thermogenic potential of the exposed brown adipocytes. These results are in line with previous studies reporting inhibitory effects of the classic androgens on BAT (33, 56). However, it should be noted that in our study higher concentrations were needed for both classical and 11-oxygenated androgens compared to previous studies. Despite the inhibitory effect of 11-oxygenated androgens on brown adipocyte differentiation, the mitochondrial mass, mitochondrial protein abundance, and mitochondrial potential remained unaltered. Similar findings have been reported previously, showing that DHT treatment in BAT explants did not affect mitochondrial DNA content and mitochondrial gene expression (33). This implies that compensatory mechanisms may be activated, which may involve other mitochondrial biogenesis regulators, such as nuclear respiratory factor 1 or mitochondrial transcription factor A (63, 64).

A previous study showed that low concentrations of T (0.1 µM) during the early stage of male mouse HB2 adipocytes differentiation decrease *Ucp1* expression (56). Similarly, the suppressive effect of DHT (10 nM-10 µM) on adipogenesis has been observed in immortalized male mouse brown adipocytes (33). However, in our study, a clear inhibitory effect on T37i cells was observed especially at high concentration (10 µM), which is markedly higher than the physiological plasma levels of ovarian androgens (0.1-2 nM) (65) and 11-oxygenated androgens (10 pM-1 nM) (66-68) in healthy women. In addition, the treated concentrations surpass the pathological ovarian androgen levels (up to 5.2 nM) in women with PCOS (69). However, studies in female mice have shown that the levels of T and its precursor androstenedione are approximately 2- to 20-fold higher in adipose tissues compared to serum concentrations (70). Therefore, the concentrations of 11-oxygenated androgens used in our study were chosen to explore a broad range of cellular responses, including tissue-accumulated androgen levels. Nevertheless, it should be noted that the highest concentration used in our study likely exceed presumed physiological and even pathophysiological androgen levels in adipose tissues. It cannot be ruled out that at this high concentration noncanonical signaling is induced. We acknowledge this as a limitation of our study.

Similar to the effects observed during differentiation, a high concentration (10 µM) of these androgens was also required to downregulate the expression of adipogenic gene markers in differentiated brown adipocytes. In contrast, previous studies report that treatment with T (0.1 µM) of differentiated stromal vascular cell-derived brown adipocytes from male mouse BAT decreases lipid accumulation and expression of brown adipocyte markers *Ucp1* and *Ppargc1a* after NE stimulation (31, 32). The differences between our findings and those of earlier publications might be attributed to intrinsic differences in the cell lines used or differences in the sex of the cells (71-73). In this study, we use T37i cells, a female-derived mouse brown adipocyte cell line, to better model the alterations in women with PCOS. Indeed, our previous study has highlighted sex differences in mouse BAT (49), with female brown adipocytes exhibiting higher activity compared to male cells (74). Therefore, higher doses of androgens may be necessary to elicit effects on female T37i cells. Furthermore, a recent study demonstrates that sexual dimorphism in BAT activity depends on Ppargc1a expression, since loss of Ppargc1a selectively impairs BAT thermogenesis in female mice, but not in males (75). The authors further revealed that estradiol (E2) treatment of BAT explants increased de novo lipogenic genes expression only in female wild-type mice, as this was blunted in female *Ppargc1a*-knockout mice, and male BAT did not respond to E2 treatment, indicating that estrogen signaling pathways are different in male and female BAT (75). Interestingly, in our study we observed that androgens downregulated *Ppargc1a* expression in T37i cells and female BAT. Collectively, these data suggest that the regulatory effects of sex steroids on BAT metabolism can be sex dependent, highlighting the importance of considering sex in biological research.

Transactivation assays using a synthetic androgen response element demonstrated that KT and KDHT exhibit potencies and efficacies comparable to T and DHT, respectively, in activating the androgen receptor (AR) (76, 77). However, unlike T, which can be converted to E2 (known to activate brown adipocyte activity (78)), KT cannot undergo this conversion by aromatase (79). Additionally, 11-oxygenated androgens are metabolized at a slower rate than ovarian androgens in prostate cancer cells (77, 80). Therefore, the 11-oxygenated androgens might exert prolonged androgenic effects in inhibiting adipogenesis compared to ovarian androgens. However, our findings suggest that 11-oxygenated androgens induced a milder effect on brown adipocyte differentiation compared to T and DHT, with KDHT showing the weakest effect. Similarly, in our in vivo study, DHT was more potent than KT and KDHT in regulating gene expression in both BAT and iWAT. A possible explanation for the different effects between the 11-oxygenated androgens and the ovarian androgens is the role of the enzyme 11β-hydroxysteroid dehydrogenase type 1 (HSD11B1) in 11-oxygenated androgen production. This enzyme, highly expressed in T37i cells and BAT (results not shown), protects AR activation from androgen excess by diminishing the androgenic potency of KT and KDHT (81). In vivo and ex vivo studies of female human adipose tissue have demonstrated that HSD11B1 not only limits the synthesis of 11-oxygenated androgens from their precursors, but also converts KT and KDHT into significantly less potent androgenic 11β-hydroxytestosterone (11OHT) and 11β-hydroxydihydrotestosterone (11OHDHT), respectively (81, 82), suggesting a higher metabolized rate in adipose tissue. Consequently, HSD11B1 may play a critical role in modulating the differential androgenic effects between ovarian androgens and 11-oxygenated androgens in BAT. While HSD11B1 drives the interconversion of the active 11-oxygenated androgens to their inactive forms, it also catalyzes the conversion of the inactive glucocorticoids to their active forms (cortisol in humans, corticosterone in mice). Given that glucocorticoids also suppress BAT activity (48), this yields a complex mechanism dissecting the adverse effects of 11-oxygenated androgens and glucocorticoids in vivo. It should be noted that we did not include corticosterone treatment in our studies, nor did we observe differences in *Hsd11b1* expression in the T37i cells and the studied adipose depots upon treatment with the androgens (data not shown).

Differentiation of adipocytes proceeds through the activation of 2 distinct waves of transcription factors (83, 84). The initial wave includes CCAAT/enhancer-binding protein β and δ (CEBPB and CEBPD) (85), which are induced during the early differentiation stage to start lipid droplet accumulation. The second wave is activated at the terminal differentiation phase when adipocytes attain their characteristic morphology, and involves increased expression of PPARG and CCAAT/enhancer-binding protein α (CEBPA) (86-88). Our results show that these markers were less affected by the analyzed 11-oxygenated androgens and ovarian androgens in mature cells compared to treatment during differentiation. Especially, the expression of *Cebpb* was decreased after androgen treatment during differentiation, but not upon treatment of mature adipocytes (data not shown). These findings are supported by previous studies showing that activated AR negatively regulates CEBPB expression and CEBPD transactivation in osteoblasts and prostate cancer cells (89, 90). Combined, these results suggest that androgens predominantly inhibit the early stage of brown adipocyte differentiation. This may also explain the relative mild in vivo effect of androgens on the BAT transcriptome, since BAT is primarily composed of mature brown adipocytes.

Compared to mature brown adipocytes, the more pronounced inhibitory effects of these androgens during differentiation may be also attributed to the extended duration of treatment. Consistently, our in vivo study showed that 1-week androgen treatment downregulated metabolic pathways in iWAT, an effect not observed following 1-day treatment. In contrast, 1-day androgen treatment resulted in the downregulation of BAT metabolic transcriptome, an effect not evident following 1-week treatment. The findings suggest a more transient, acute response of BAT to KT exposure, and a more sustained response in iWAT, indicating a loss of beiging in iWAT combined with potential compensatory mechanisms in BAT. However, both the 1-day and 1-week treatments employed in our study represent relatively short durations compared to the long-term androgen exposure seen in PCOS. In addition to direct effects on BAT, sex steroids could also have indirect effects on BAT activity, given that these steroids also regulate energy metabolism through central mechanisms (74). Indeed, in vivo studies have shown a critical role of AR in the brain in the development of reproductive and metabolic PCOS traits (91, 92). Therefore, it will be interesting to elucidate the long-term effects of 11-oxygenated androgens on BAT metabolism and the role of central mechanisms therein.

Although the metabolic phenotype is in general milder in normoandrogenic than hyperandrogenic women with PCOS, human studies have indicated that also normoandrogenic women with PCOS exhibit an adverse metabolic profile compared to healthy individuals (93-97), which may suggest an adrenal contribution to the phenotype. Since our results show that even short-term exposure to 11-oxygenated androgens suppresses BAT metabolism and diminishes iWAT thermogenic potential, these effects may be explained by elevated 11-oxygenated androgens, which are not routinely measured in women with PCOS, and whose serum levels remain relatively stable with aging and are more abundant than classic androgens in postmenopausal women (67). To further validate this hypothesis, it would be interesting to perform long-term treatment studies with 11-oxygenated androgens, preferably in ovariectomized animal models, to eliminate potential confounding effects of ovarian androgens.

In conclusion, our study shows that 11-oxygenated androgens inhibit brown adipocyte differentiation in vitro, and impair both BAT metabolism and WAT thermogenic potential in vivo, underscoring their contribution to the metabolic dysfunction observed in women with PCOS and other hyperandrogenic conditions, such as congenital adrenal hyperplasia (68). Further research into the molecular mechanisms through which 11-oxygenated androgens regulate BAT is needed to fully decipher the role of 11-oxygenated androgens in the pathophysiology of hyperandrogenic conditions, including PCOS. Since the circulating androgen pool consists of a mixture of these androgens, it would be interesting to determine their combined and long-term effects on BAT activity and systemic metabolism.

## Disclosures

J.A.V. has received royalties from anti-Müllerian hormone assays, paid to the institute/laboratory with no personal financial gain. The other authors have nothing to disclose.

## Data Availability

The datasets generated during and/or analyzed during the current study are not publicly available but are available from the corresponding author on reasonable request.

## Abbreviations

AKR1C3: aldo-keto reductase 1C3
AR: androgen receptor
BAT: brown adipose tissue
cDNA: complementary DNA
DCC: dextran-coated charcoal
DEGs: differentially expressed genes
DHT: dihydrotestosterone
E2: estradiol
ES: enrichment score
EtOH: ethanol
FBS: fetal bovine serum
FDR: false discovery rate
GSEA: Gene Set Enrichment Analysis
HSD11B1: 11β-hydroxysteroid dehydrogenase type 1
IR: insulin resistance
iWAT: inguinal white adipose tissue
KDHT: 11-ketodihydrotestosterone
KEGG: Kyoto Encyclopedia of Genes and Genomes database
KT: 11-ketotestosterone
mRNA: messenger RNA
NE: norepinephrine
PBS: phosphate-buffered saline
PCOS: polycystic ovary syndrome
PCR: polymerase chain reaction
RNAseq: RNA sequencing
T: testosterone
T3: triiodothyronine
UCP1: uncoupling protein 1
WAT: white adipose tissue
